# Contiguous Burst Fractures of the Lumbar Spine

**DOI:** 10.7759/cureus.63313

**Published:** 2024-06-27

**Authors:** Sai Preeth, Vijayanand B, Rishab C

**Affiliations:** 1 Department of Orthopaedics and Traumatology, SRM Medical College Hospital and Research Centre, Chennai, IND

**Keywords:** spinal canal, multiple, contiguous, lumbar, burst fracture

## Abstract

Burst fractures of vertebrae are usually caused by high-energy axial compression force, mostly caused by fall from height or road traffic accidents. They frequently occur at the thoracolumbar junction mostly requiring surgery. Contiguous burst fractures involving multiple lumbar vertebrae are uncommon. This case is a male in his early 40s presented with low back pain and weakness of lower limbs following an injury sustained during a road traffic accident. Clinically, the patient had a bilateral foot drop. On radiological evaluation, he was diagnosed to have L3 and L4 burst fractures with spinal canal occlusion. He underwent posterior stabilization from L2-L5 and decompression at the L3-L4 level. At one-year follow-up, the patient was pain-free with complete neurological recovery. Contiguous lumbar spine burst fractures are very rare in occurrence. Though burst fractures are managed surgically to provide stability, the surgical approaches depend on the individual fracture pattern, degree of spinal canal occlusion, and neurological status.

## Introduction

Burst fractures of vertebrae are usually caused by high-energy trauma due to axial compression force, mostly caused by a fall from height or road traffic accidents. The treatment for burst fractures depends on the individual fracture pattern and neurological status of the patient. When two or more spinal columns are damaged, Denis deems the burst fracture to be unstable [1]. However, there is still debate regarding burst fracture stability criteria and available treatments. It is generally agreed that all acute thoracolumbar fractures should first be treated as unstable [2]. Because the lumbar spine is flexible, the occurrence of burst fracture at the lumbar level is thought to be rare compared to the thoracic spine. Contiguous fractures involving the cervical and thoracic spine have been reported. To date, only three cases have been documented regarding contiguous lumbar burst fractures, out of which one of those cases was due to seizure activity.

## Case presentation

A male in his early 40s was brought to the emergency department with a history of skid and fall from a two-wheeler sustaining an injury to the lower back. He had tenderness over the lumbar spines and multiple abrasions involving the entire back. His ankle and toe dorsiflexion were of grade 0/5 on the right side and 2/5 on the left side. He also had a sensory deficit at the L4 and L5 dermatomes on both sides. However, there was no bladder or bowel incontinence.

**Figure 1:**
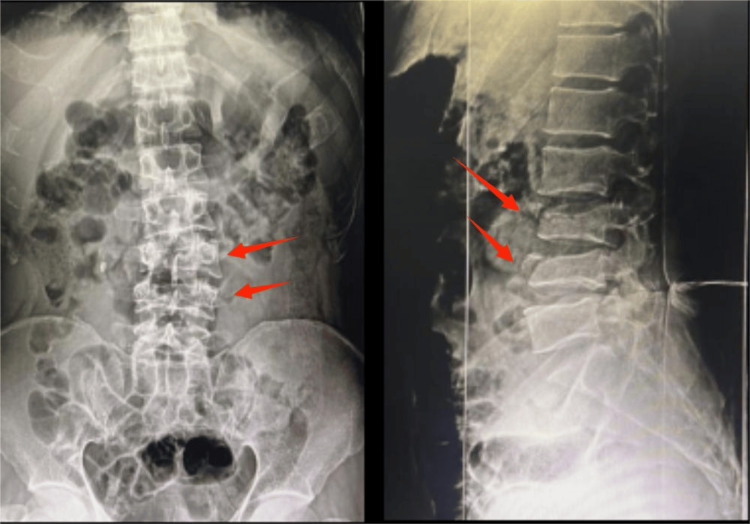
Anteroposterior and lateral views of X-ray of lumbosacral spine showing L3 and L4 burst fractures

**Figure 2:**
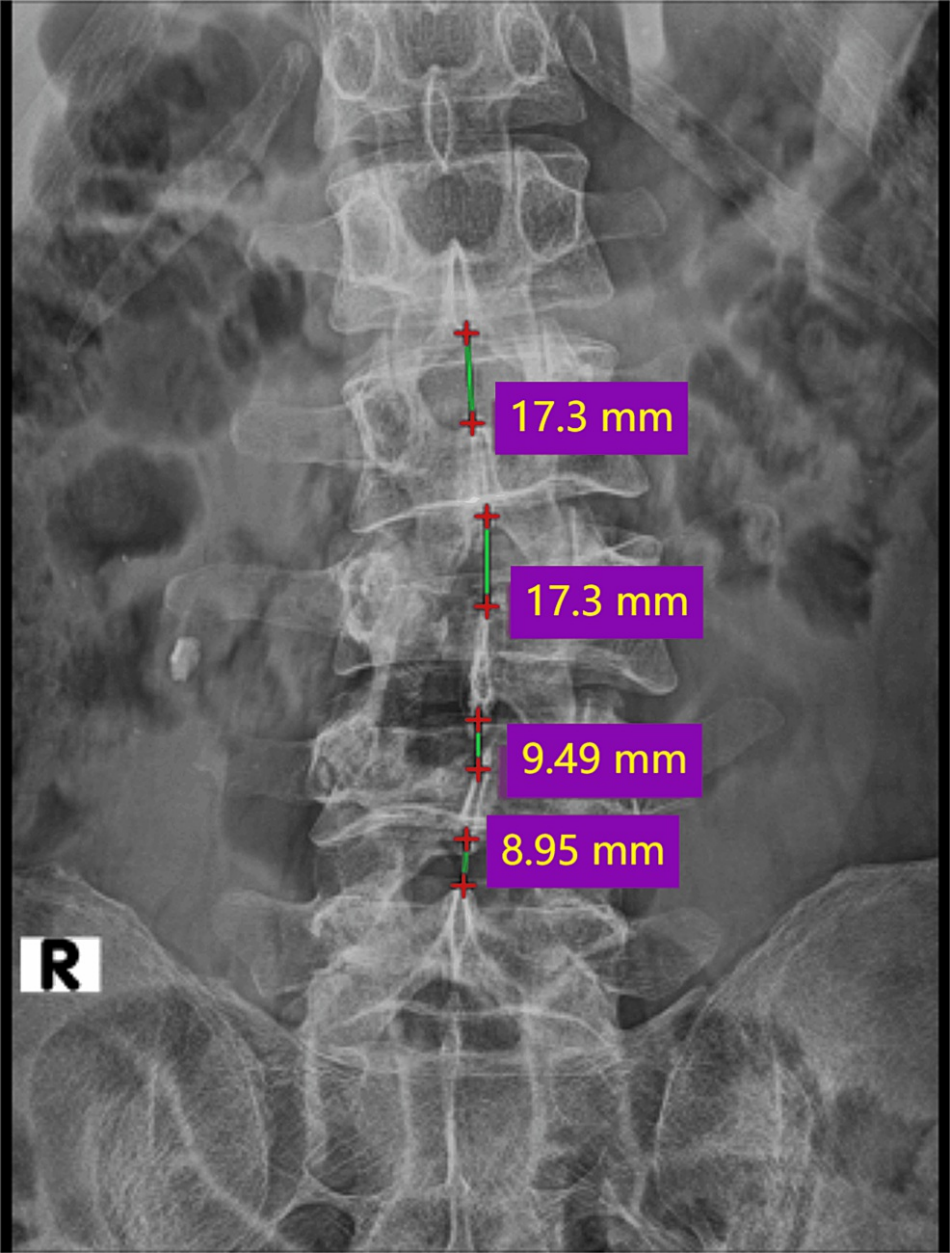
Decreased interspinous distance between L3, L4 vertebra and L4, L5 vertebra when compared to interspinous distance of uninjured vertebra between L1, L2 and L2, L3

**Figure 3:**
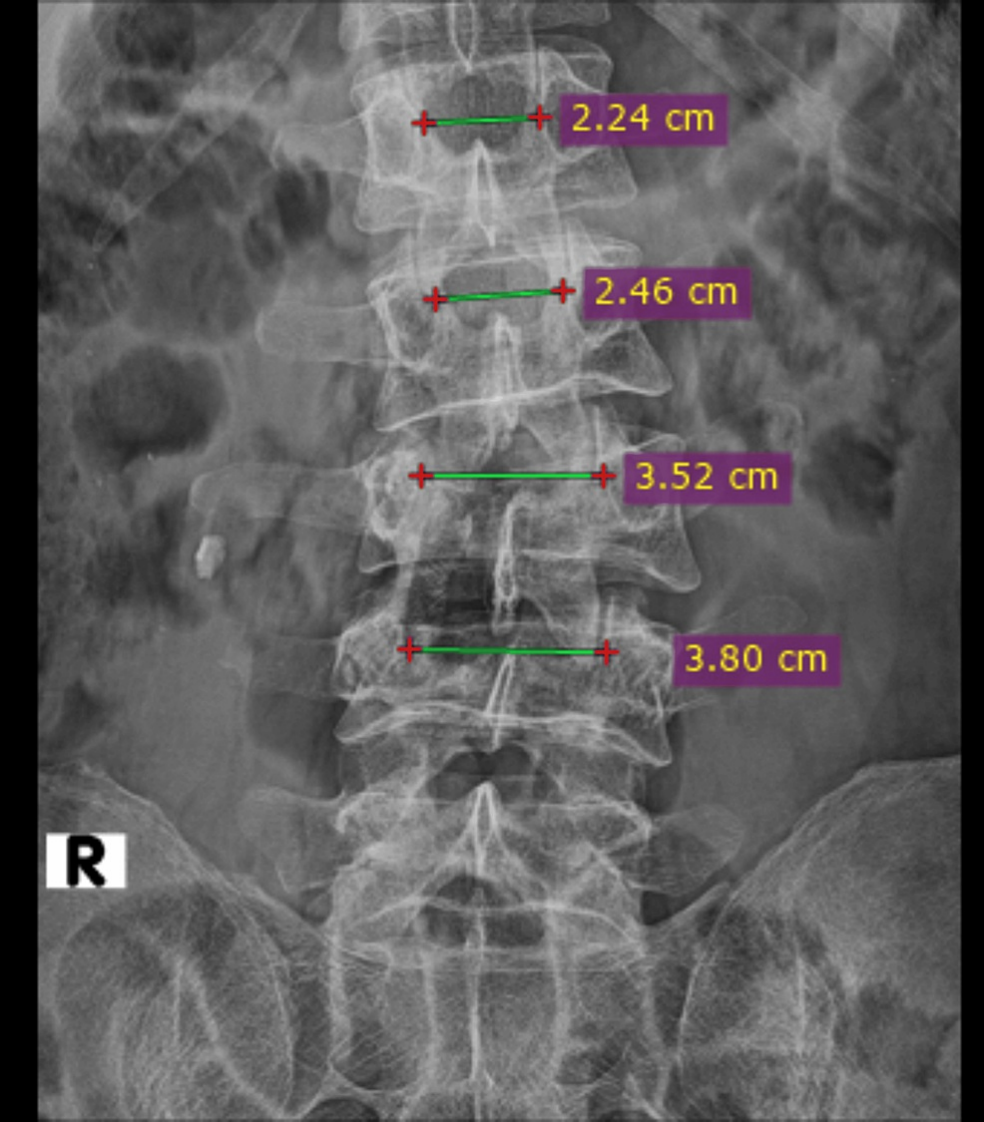
L3 and L4 vertebrae showing increased inter-pedicle distance when compared to the uninjured L1 and L2 vertebrae

**Figure 4:**
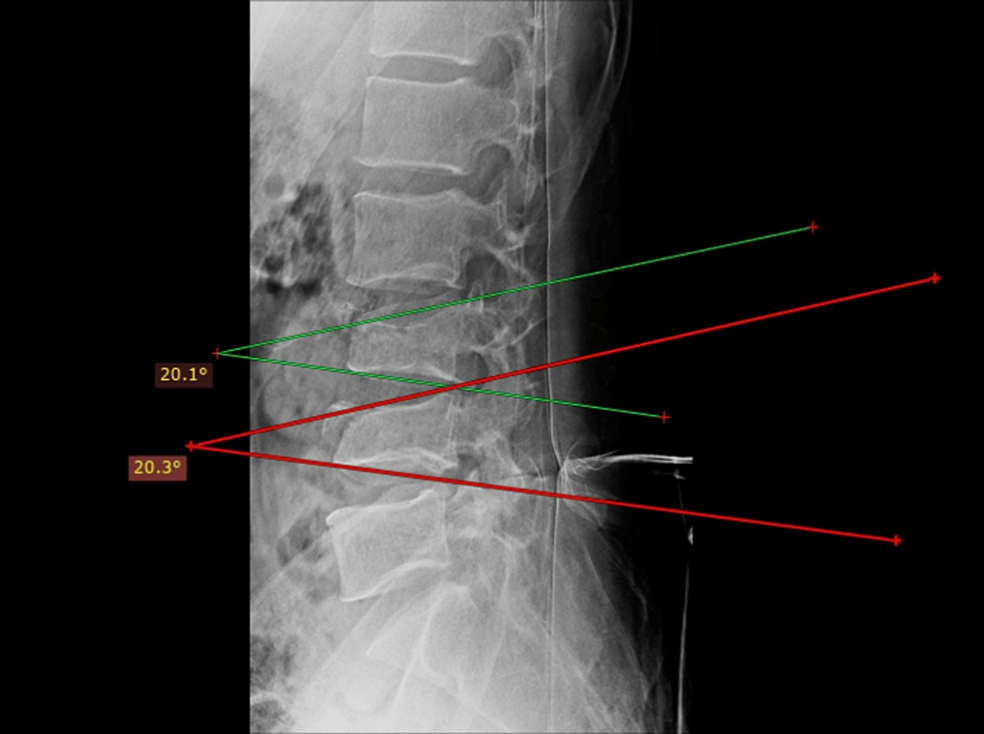
L3 vertebra and L4 vertebra showing decreased kyphotic angle

**Figure 5:**
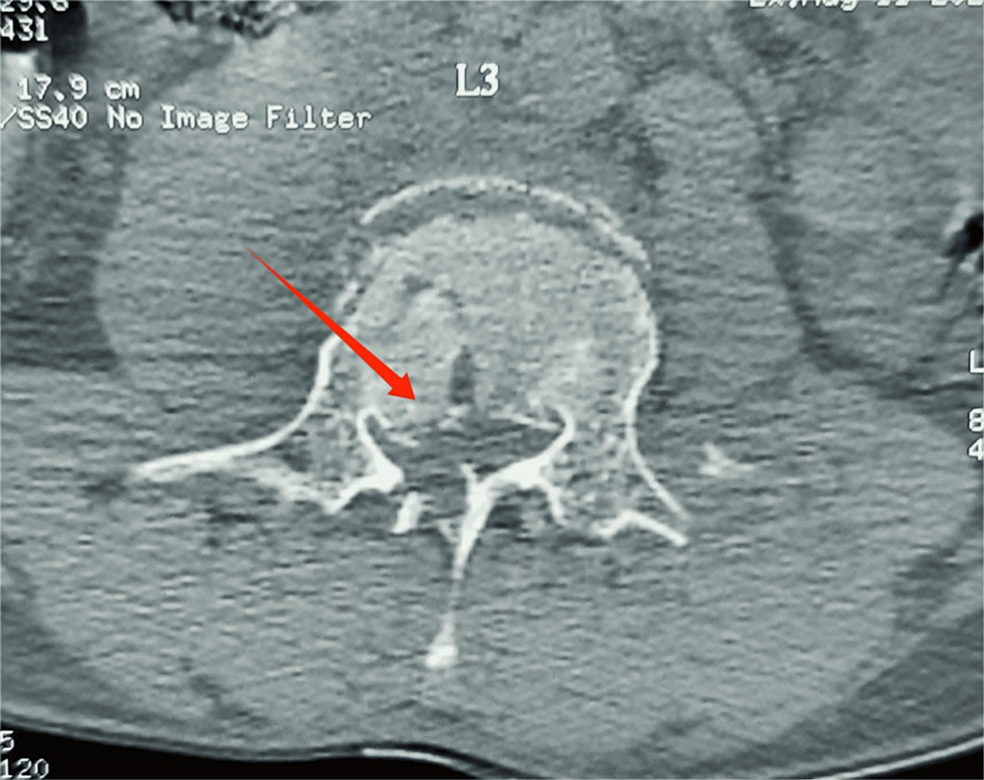
CT showing L3 burst fracture with retropulsion of fragment CT, computed tomography

**Figure 6:**
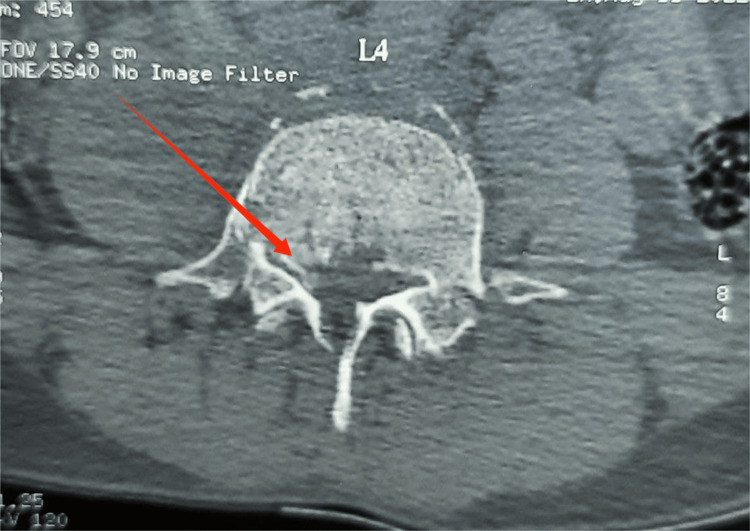
CT showing L4 burst fracture with retropulsion of fragment

**Figure 7:**
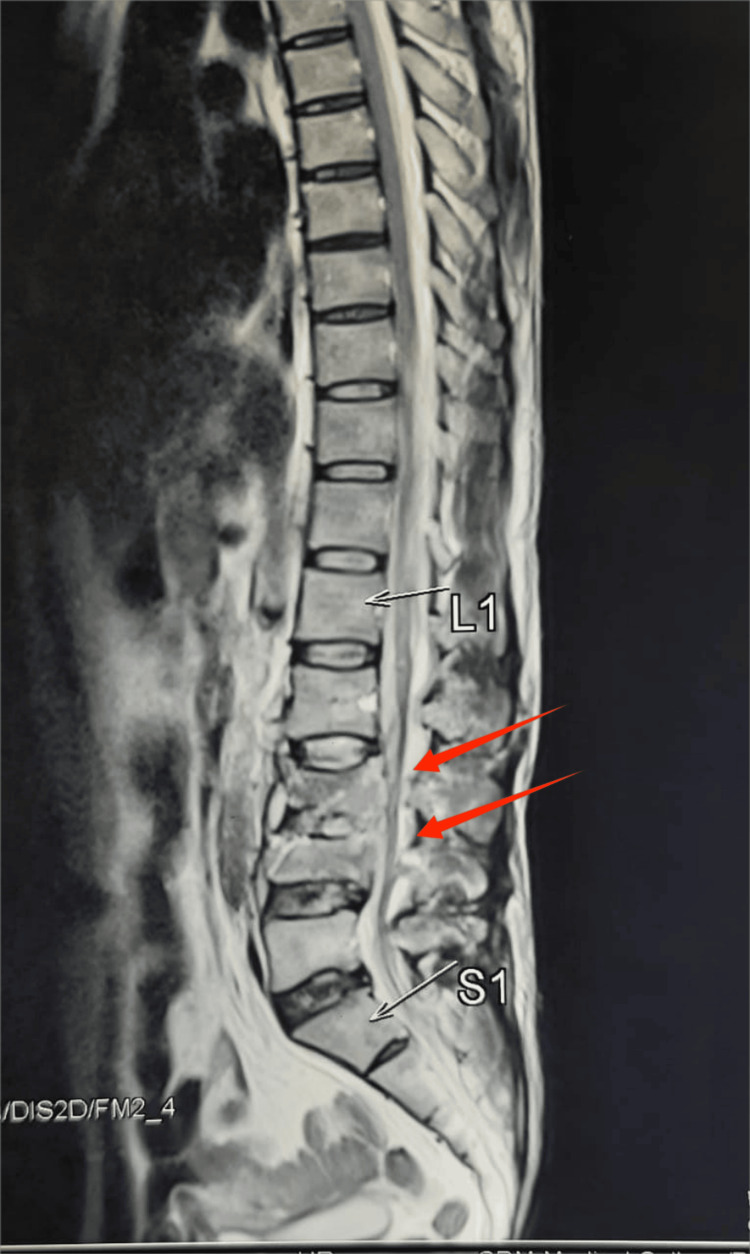
MRI showing L3 and L4 burst fractures with <50% neural canal occlusion MRI, magnetic resonance imaging

**Figure 8:**
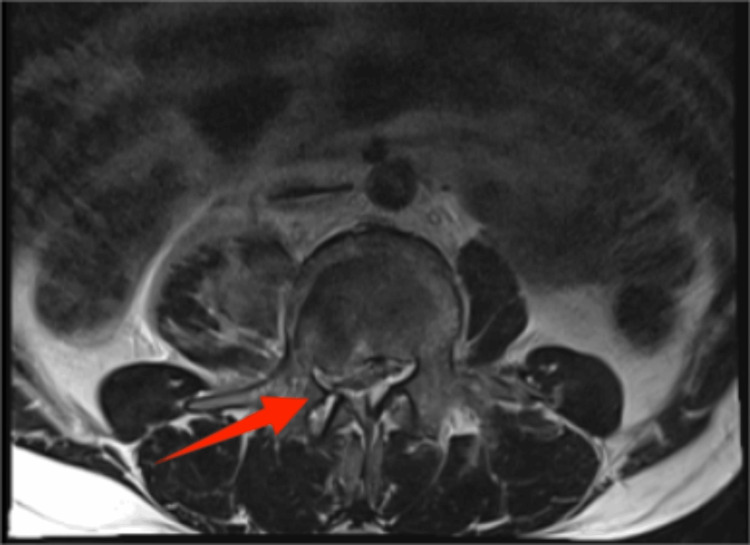
MRI axial image showing L3 vertebral level MRI, magnetic resonance imaging

**Figure 9:**
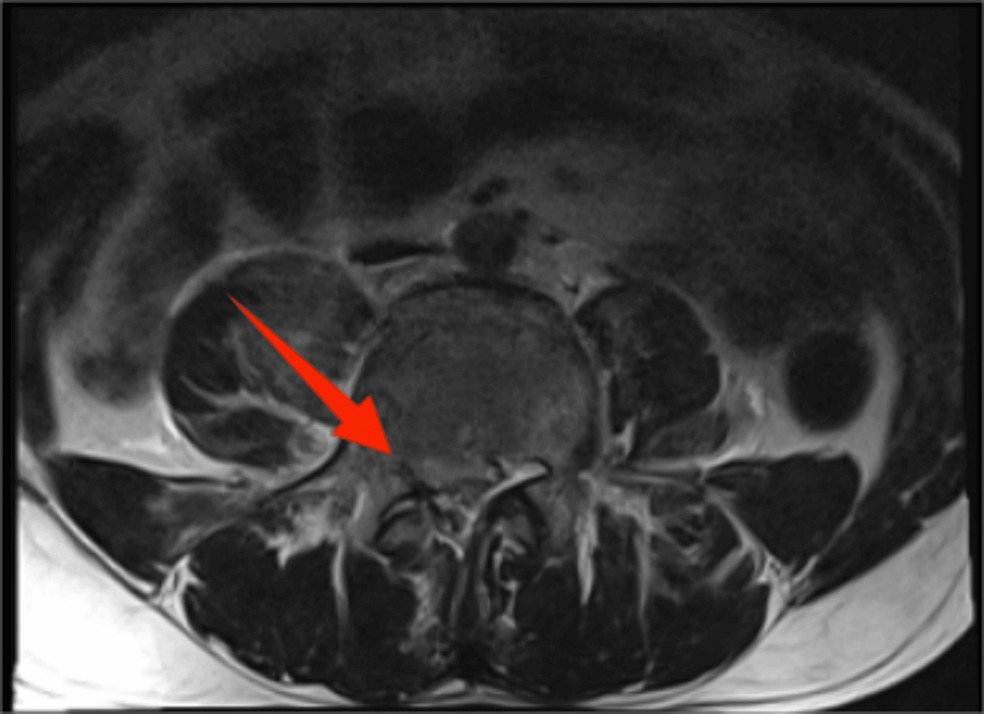
MRI axial image showing L4 vertebral level MRI, magnetic resonance imaging

Plain radiographs of lumbosacral spine anteroposterior and lateral views (Figure 1) revealed decreased vertebral height at L3 and L4, decreased inter-spinous distance (Figure 2), and increased inter-pedicular distance at L3 and L4 (Figure 3), with local kyphosis involving those levels (Figure 4). Computed tomography (CT) of the lumbar spine revealed burst fracture of L3 and L4 with retropulsion of fragments at both levels (Figures 5, 6). Magnetic resonance imaging (MRI) showed stenosis of the spinal canal at L3-L4 levels with <50% occlusion (Figures 7, 8, 9). His thoracolumbar injury classification and severity score (TLICS) was six [3]. According to the AO classification of thoracolumbar injuries, for L3 and L4 it would be A4 N1 M1 [4].

**Figure 10:**
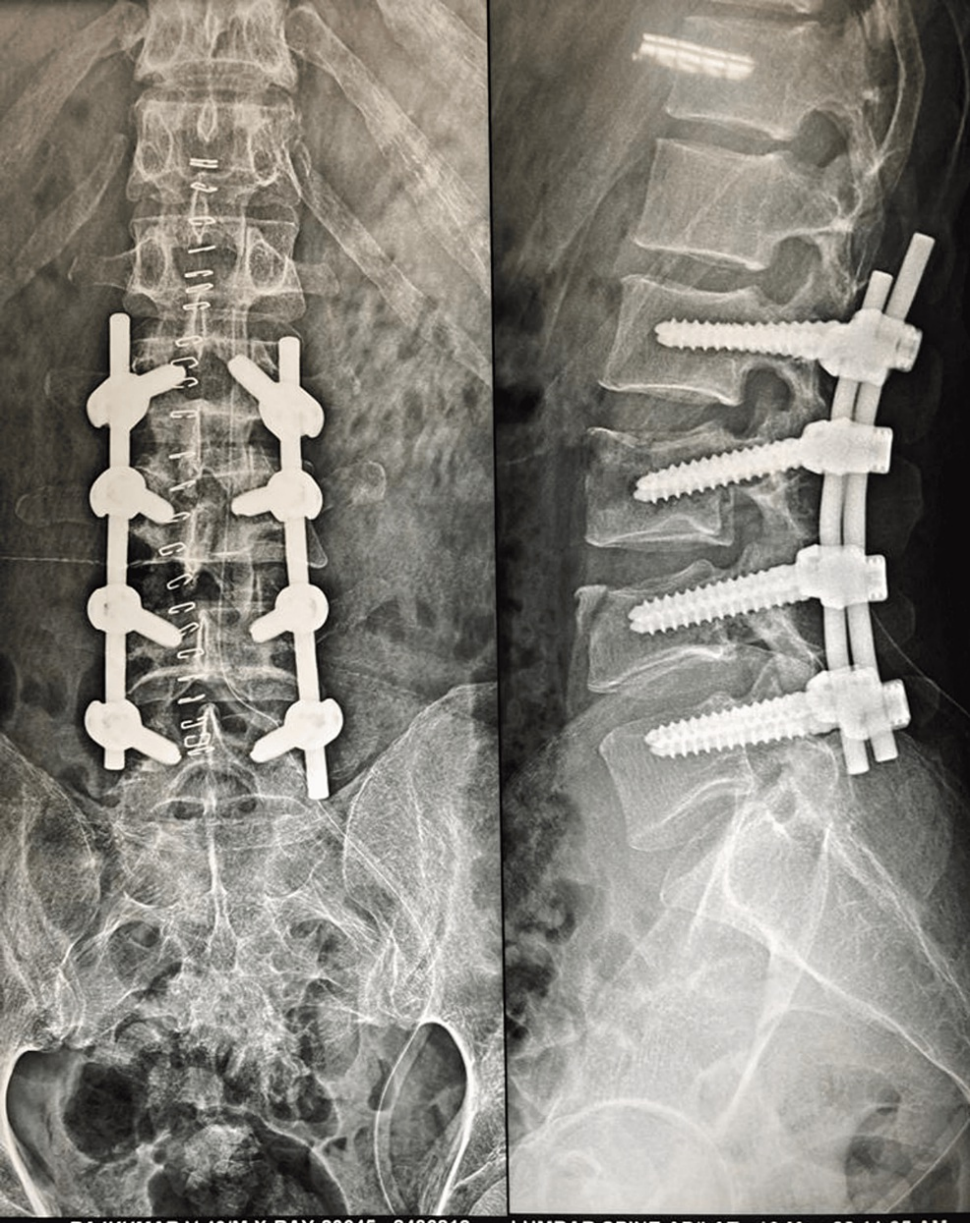
L2 to L5 posterior stabilization with L3-L4 hemi-laminectomy (immediate post-operative image)

The patient underwent posterior decompression and instrumented stabilization from L2 to L5 in the prone position under general anesthesia over a radiolucent Wilson frame. Through an open mid-line approach, pedicle screw fixation was done from L2 to L5 levels using the intersection technique. Bilateral hemi-laminectomy at L3 and L4 levels was done to decompress the neural structures. The adequacy of decompression was ensured with nerve root probes. Epidural hematomas found were washed away and the wound closed in layers. The patient was mobilized with lumbosacral orthosis and a foot drop splint on both sides in the immediate post-operative period. He had an uneventful post-operative period and wound healing. Post-operative radiographs showed improvement in vertebral height and restoration of normal lumbar lordosis (Figure 10).

**Figure 11:**
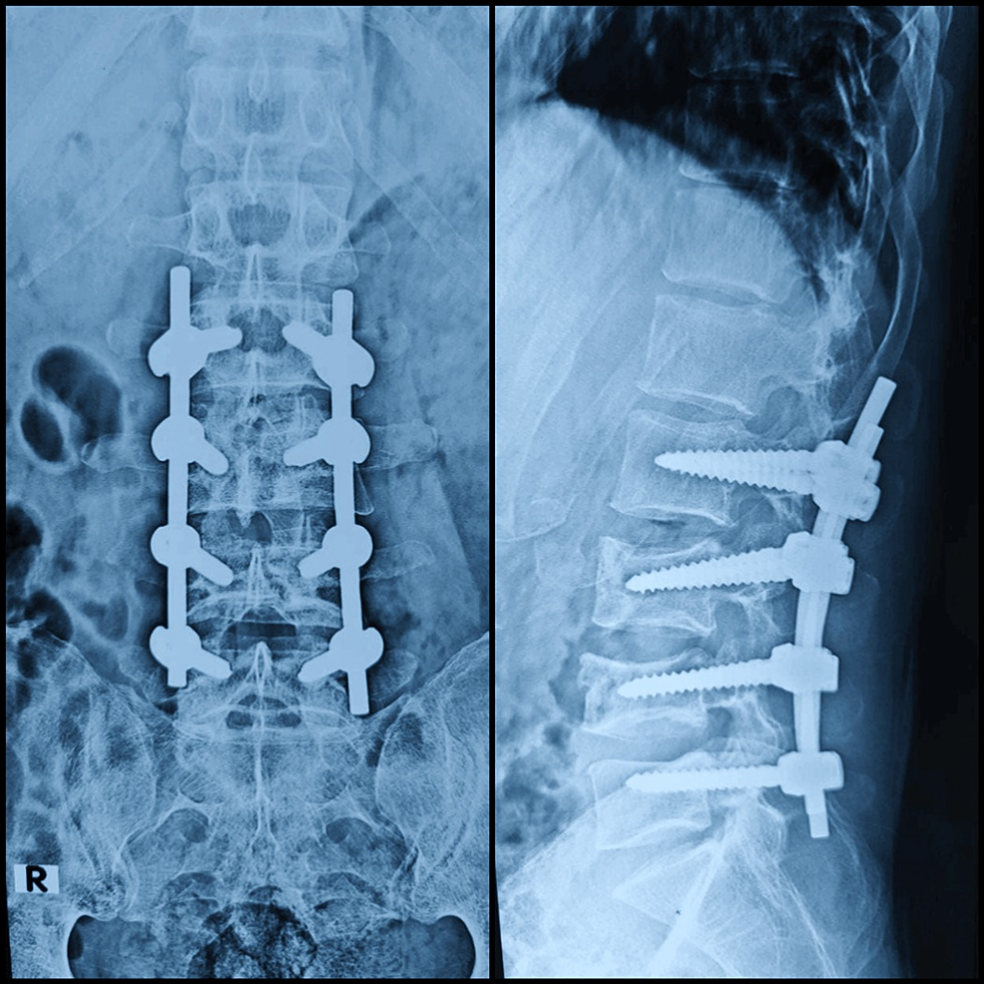
One-year post-operative X-ray of lumbosacral spine - anteroposterior view and lateral view

**Figure 12:**
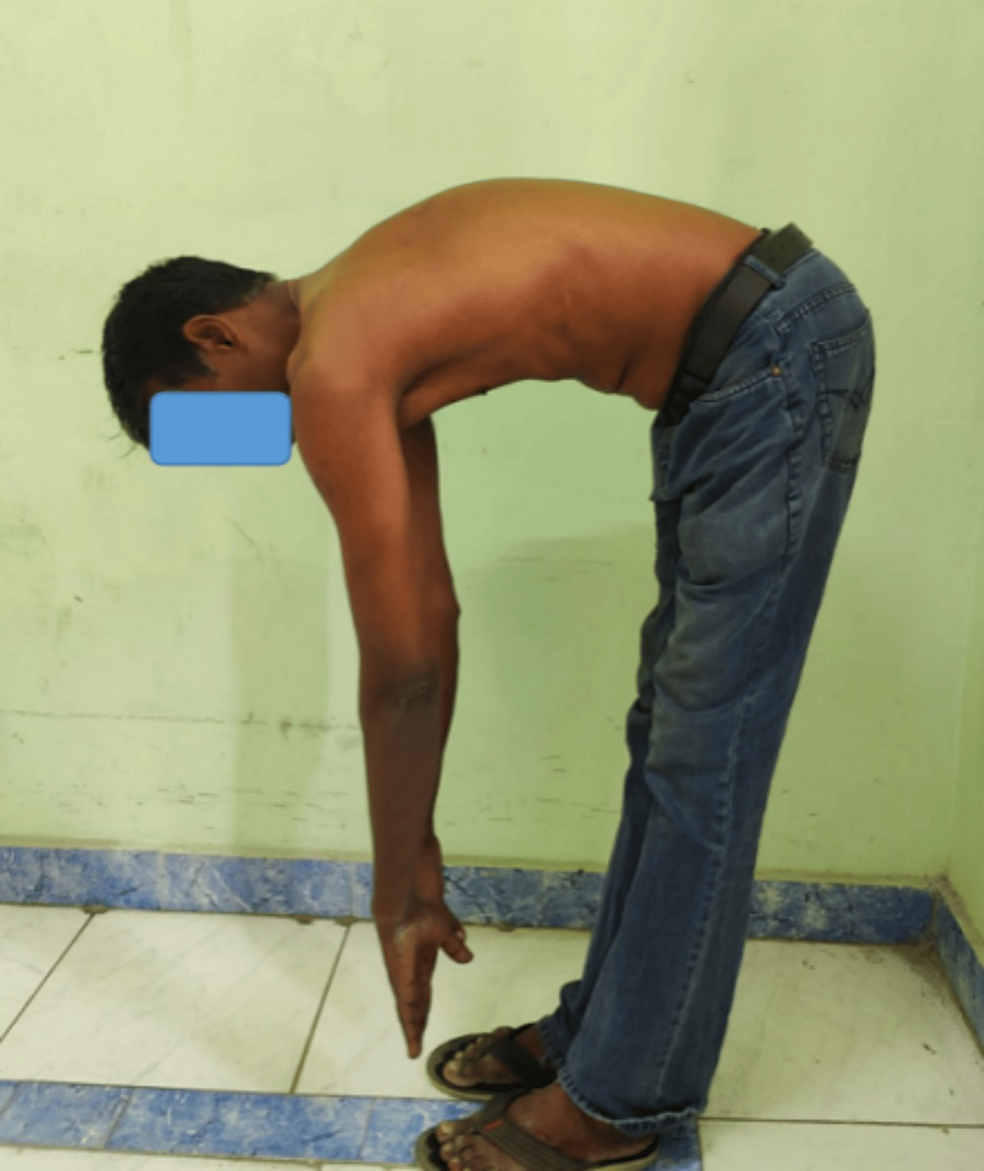
One-year post-operative active flexion

**Figure 13:**
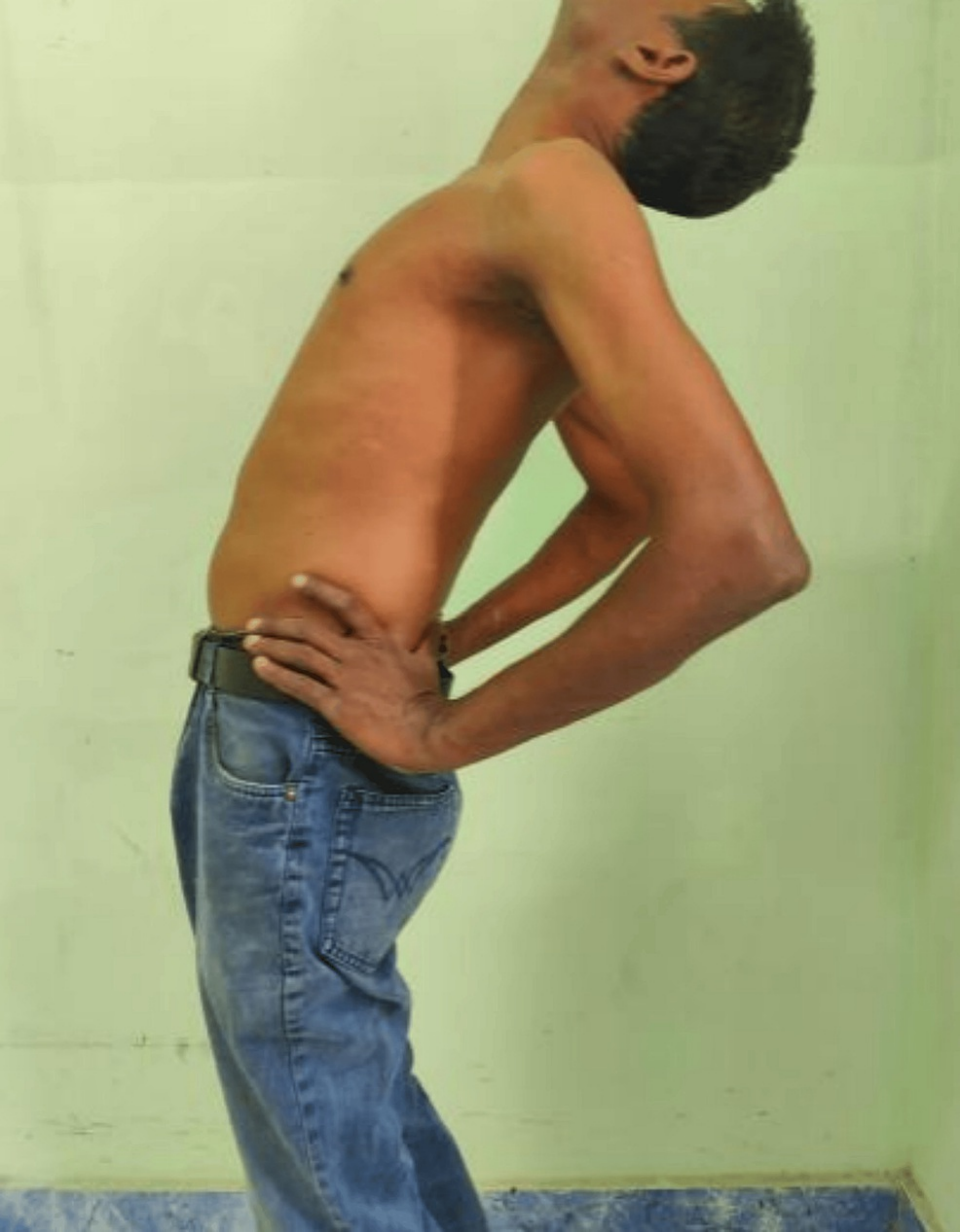
One-year post-operative active extension

At the six-month follow-up, the patient was found to have complete neurological recovery and also resumed his profession as an electrician with difficulty lifting weights from the floor. At one year, he was independently performing in his profession. Follow-up radiographs taken at one year showed no late collapse of the vertebrae or loosening of implants (Figure 11). The patient showed good spinal movements at the end of the first year (Figures 12, 13).

## Discussion

Burst fractures are common in the thoracolumbar region, which is the transition zone between the rigid thoracic level and the flexible lumbar level. The three-column theory by Denis et al. divides the vertebral column into anterior, middle, and posterior columns [1]. The columns are divided based on their bio-mechanical stability following traumatic injury. Denis also postulated that instability occurs when two contiguous columns are injured. About 90% of all spinal fractures occur between levels D11 and L4, while burst fractures account for 14-17% with 26% of patients having neurological deficits. Denis had classified burst fractures as impairment of the middle column with displacement or rotation of the posterior cortex of the vertebral body [1].

There are many cases reported of non-contiguous multiple-level burst fractures involving the cervical and thoracic vertebrae. As such, burst fractures of the lower lumbar spine are rare due to its flexible biomechanics. To date, only three case reports have been documented on contiguous lumbar burst fractures, out of which two have a history of trauma and one case report is due to tonic-clonic seizures [5-7].

Treatment includes stabilization of the injured level with decompression for neural canal compromise, the approaches being anterior, posterior, or combined. In a prospective study, Korovessis et al. compared the outcomes of posterior "short-segment" trans-pedicular fixation with combined anterior-posterior surgery and found that, despite the placement of intermediate screws in the fractured vertebra, the resulting construct did not stop the average 5° loss of correction at the final evaluation [8]. Due to anterior column disruption, there can be implant failure, pseudo-arthrosis, etc. A study done in 2006 by Korovessis et al. compared anterior combined with posterior approach with the posterior short segment approach and found the combined approach to be superior [8].

In a study done by Wood et al., in 2005, the results were similar in both the anterior and posterior approaches. However, this was performed on patients without neurological deficits [9]. A study done by Limb et al., in 1995, showed that neurological deficits happen due to the velocity of injury and not bone fragments in the canal. Decompressing the canal or not did not correlate with neurological recovery [10]. There are many studies that found no statistical difference between conservative and operative treatments in patients with burst fractures but intact neurological status [9,11,12].

Dhillon et al. reported minimally invasive posterior stabilization and anterior hemi-corpectomy of L2 and L4 and fusion for a contiguous three-level burst fracture involving L2, L3, and L4 vertebrae [5]. They suggested anterior decompression if the neural canal occlusion was >50%. Since they used both anterior and posterior stabilization, short-segment fixation was followed. Long-segment posterior stabilization though found to be a stable construct, can reduce the mobility of the spinal segment, whereas short-segment fixation maintains mobility. In our case report, the patient had a contiguous, two-level unstable burst fracture of the lower lumbar spine with neurological impairment and, hence, we did posterior stabilization from L2 to L5 with hemi-laminectomy L3-L4, as there was <50% neural compromise.

## Conclusions

Burst fractures commonly occur at the thoracolumbar junction as it is a transition zone between the rigid thoracic spine and mobile lumbar spine. Though burst fractures are regarded as unstable, not all fractures require surgery. The patient in this report underwent surgery as it was a contiguous burst fracture with neurological deficit, who showed complete recovery at the end of one year. Surgical approaches for burst fracture should be individualized depending on fracture pattern, spinal canal occlusion, and neurological status of the patient. Anterior and posterior approaches for stabilization might be needed if spinal canal occlusion is more than 50%.
